# Thinking Out of the Box: On the Ability of *Zea mays* L. to Biotrasform Aflatoxin B1 Into Its Modified Forms

**DOI:** 10.3389/fpls.2020.599158

**Published:** 2021-01-12

**Authors:** Laura Righetti, Enrico Rolli, Luca Dellafiora, Gianni Galaverna, Michele Suman, Renato Bruni, Chiara Dall’Asta

**Affiliations:** ^1^Department of Food and Drug, University of Parma, Parma, Italy; ^2^Department of Chemistry, Life Sciences and Environmental Sustainability, University of Parma, Parma, Italy; ^3^Barilla G.R. F.lli SpA, Advanced Laboratory Research, Parma, Italy

**Keywords:** modified mycotoxins, plant resistance, xenobiotics, *Aspergillus*, pathogen fungi

## Abstract

While aflatoxin metabolism in animals has been clarified, very limited information is so far available on the possible biotransformation occurring in plants. Therefore, this work aimed at investigating whether AFB1 metabolites could occur in field-grown infected maize and the putative role of *Zea mays* L. metabolism in their production. For such scope, asymptomatic *in vitro*–grown plantlets and *in silico* evaluations of plant transforming enzymes were used to pinpoint how plants may handle these compounds. Our data demonstrated the role of maize plants in the production of Phase I hydroxylated aflatoxins, including, among others, AFM1, AFM2, and aflatoxicol, and suggest that plant cytochromes may be involved in this biotransformation of AFB1.

## Introduction

Aflatoxins, on a worldwide scale, are the most well-known and studied mycotoxins in food and feed and must be regarded as a major food safety and food security threat (Hussein and Brasel, 2001; Marin et al., 2013; Kumar et al., 2017). Especially in low- and middle-income countries, it is estimated that approximately 4.5 billion people are chronically exposed to largely uncontrolled amounts of aflatoxin that severely affect the health status (Williams et al., 2004). Aflatoxin contamination indeed may have an impact on food commodities, mainly grains, causing relevant economic losses and representing a serious concern for human and animal health. Aflatoxin-producing fungi, mainly *Aspergillus flavus* and *Aspergillus parasiticus*, are found in areas with a warm, humid climate and may infect crops at both preharvest and postharvest stage. Climate change is strongly related with an increasing trend of aflatoxin occurrence in crops, mainly maize, as reported by Battilani et al. (2016).

Besides the four main aflatoxins (AFB1, AFB2, AFG1, and AFG2), aflatoxinogenic fungi may also produce other structurally related substances such as versicolorin, and sterigmatocystin. In addition, hydroxylated metabolites, among them the most relevant AFM1 or the aflatoxicol (AFL), are known to be formed in animals, accumulated in tissues and fluids, or transferred to milk as a consequence of liver Phase I biotransformation (Flores-Flores et al., 2015; Braun et al., 2020).

While aflatoxin metabolism in animals has been clarified, and major metabolites as well as conjugates have been structurally elucidated (Deng et al., 2018), very limited information is so far available on the possible biotransformation occurring in plants. Nonetheless, AFM1, together with other hydroxylated aflatoxins, has been occasionally detected in highly contaminated crops grown under field conditions over years, without the scientific community really questioning its origin.

To our best knowledge, the first reports on AFM1 in corn dated back in 1975, when the occurrence of AFM1 in stored and freshly harvested highly contaminated corn was described (Shotwell et al., 1976). Its presence in peanuts and pistachio nuts was already attested (Waltking, 1975) and confirmed later on (Huang et al., 2010). In their article, Shotwell et al. (1976) stated that, because of the very low concentration compared to AFB1, “the presence of M1 in corn has little practical significance.”

Later on, Saito et al. (1984) described the presence of AFL, in pistachio nuts and corn. Its formation was ascribed to the AFL-producing ability of *A. flavus* itself (Nakazato et al., 1985, 1990), as well as to the conversion ability of several coinfecting fungi (Nakazato et al., 1985). However, in the following years, the availability of highly specific analytical methods mainly based on immunoaffinity column clean-p, and the urgency to focus the monitoring plans on the main and most dangerous aflatoxins meant that the possible presence of AFM1 and other hydroxylated metabolites in crops was totally neglected. In particular, no clear and robust explanation has been provided whether these hydroxylated aflatoxins are produced by co-occurring microorganisms, by the plant metabolism itself, or by other causes.

In more recent years, the advent of multitoxin methods and the lowering of detection limits allowed for a larger and less targeted monitoring of occurring mycotoxins, thus offering the opportunity to find also unexpected contaminants. In particular, a number of studies performed on food from rural areas in Africa and in other low-income countries listed AFM1, AFL, and other hydroxylated forms, among the co-occurring mycotoxins found in crops and traditional meals, where the fungal infection and the subsequent aflatoxin accumulation were high (Ezekiel et al., 2012a,b; Adetuniji et al., 2014; Chala et al., 2014; Matumba et al., 2015; Ojuri et al., 2018, 2019). AFM1 was also detected in roasted peanut from Sierra Leone (Sombie et al., 2018).

However, as made by authors in the previous decades, the origin of AFM1 and other hydroxylated aflatoxins were not specifically addressed by any of the aforementioned articles, whereas some authors merely suggested that AFM1 was formed in the field or during storage by fungal, bacterial, or insect metabolism, neither demonstrating nor specifically investigating this statement.

While both bacterial and fungal metabolisms may biotransform AFB1 into AFM1 (Mann and Rehm, 1976, 1977; McCormick, 2013), nothing is known so far about insects’ capability. In addition, it should be considered that AFM1 can be produced by *A. flavus* itself starting from AFB1, although at a very low conversion rate (i.e., AFM1–AFB1 ratio: 0.0025–0.01, according to Hesseltine et al., 1970) and thus in almost negligible amounts.

To the best of our knowledge, none of the previous studies has taken into consideration the possible role of the plant “green liver” in transforming AFB1 into its hydroxylated forms (Sandermann, 1994). The metabolic machinery that plants have developed to cope with xenobiotics has been largely described for many manmade chemicals (Bártíková et al., 2015; Del Buono et al., 2020). It consists of a cascade of enzymatic reactions broadly categorized as Phase I and Phase II metabolism, aimed at increasing the polarity of the xenobiotic compound mainly through the conjugation with polar moieties (Sandermann, 1994; Huber et al., 2012; Chen et al., 2016). The resulting metabolites may then follow different routes, including the deposition in the apoplast, cell wall bounding, or vacuolar segregation, often with the final aim of their expulsion through organ senescence. This concept is actually at the basis of the formation of the so-called masked mycotoxins, which are modified forms of mycotoxins originated from plant biotransformation (Berthiller et al., 2005).

Great efforts have been spent over the last decade to elucidate in various crops the formation, significance, and occurrence of modified *Fusarium* mycotoxins, mainly those related to deoxynivalenol and zearalenone (Berthiller et al., 2013). Several public bodies, among them the European Food Safety Authority, have been recognized that modified mycotoxins pose a significant risk in food and feed as they may increase the overall burden of concern (Steinkellner et al., 2019).

Despite the bulk of research about *Fusarium* masked mycotoxins, only two studies have investigated the possible occurrence of conjugated (i.e., “masked”) aflatoxins (Zivoli et al., 2014; Vidal et al., 2018). Unfortunately, both studies have used indirect methods for detection, and thus neither provided any putative identification nor suggested the chemical structure for the hypothesized conjugated forms. Annotation and characterization of unknown metabolites, indeed, require the use of techniques able to provide structural information, i.e., high-resolution mass spectrometry (HRMS) and nuclear magnetic resonance, and the setup of hypothesis-driven experiments, focused at investigating the biotransformation mechanism at a molecular level.

In the view of better understanding the biological pathways involved, we have recently set up an *in vitro* model based on micropropagation to explore the formation of modified form of several *Fusarium* mycotoxins in durum wheat (Righetti et al., 2017, 2019, 2020; Rolli et al., 2018). The model, although still far from the real in field scenario, proved to represent a valuable tool to elucidate the plant biosynthetic potential for masked mycotoxins formation and to identify possible metabolites to be further targeted and validated in crops.

Therefore, this work is aimed at confirming whether any of the main hydroxylated AFB1 metabolites could occur in field-grown infected maize and at the investigation of the putative role of *Zea mays* L. metabolism in their production. For such scope, asymptomatic plantlets grown under controlled conditions and *in silico* evaluations were used to pinpoint how plants may handle these compounds. Specifically, a previously validated three-dimensional (3D) molecular modeling approach (Dellafiora et al., 2020) was applied to study the interaction of AFB1 with potential plant transforming enzymes from a molecular standpoint.

## Materials and Methods

### Chemicals and Reagents

Aflatoxin B1 solid standard and aflatoxin mix containing AFB1 and AFB2 (2,000 μg mL^–1^ in acetonitrile), AFG1 and AFG2 (500 μg mL^–1^ in acetonitrile), and AFM1 (solution in acetonitrile 500 μg mL^–1^) were obtained from Biopure (Romer Labs, Tulln, Austria). AFM2 and AFL (100 μg) were from Tebu-Bio Srl (Magenta, Italy).

Afla M1 HPLC^TM^ immunoaffinity columns were purchased from Vicam (Milford, MA, United States).

High-performance liquid chromatography (LC)–grade methanol, acetonitrile, and acetic acid were purchased from Sigma–Aldrich (Taufkirchen, Germany); bidistilled water was obtained using Milli-Q System (Millipore, Bedford, MA, United States). Mass spectrometry (MS)–grade formic acid from Fisher Chemical (Thermo Fisher Scientific Inc., San Jose, CA, United States) and ammonium acetate (Fluka, Chemika-Biochemika, Basil, Switzerland) were also used.

### Plant Material

Maize samples (*N* = 15) were collected over 2016/2017 growing season in a varietal field located in Emilia-Romagna region (Italy). The full-grown ears were dried at 60°C at ca. 14% humidity immediately after manual harvest and shelled using an electric sheller. Kernels were ground using a laboratory mill (A11 Basic Analytical Mill, IKA, Stauffen, Germany), passed thought a 1-mm sieve to obtain a whole meal and stored at +4°C until the analysis.

### Plantlets Growth Conditions and AFB1 Administration

Maize (*Z. mays* L.) hybrid (FAO class 300) caryopses were soaked in 70% (vol/vol) ethanol for 5 min and then rinsed three times in sterile distilled water. Surface disinfection was performed with 2.5% (vol/vol) sodium hypochlorite for 50 min under vacuum (−15 inch of Hg), followed by six washes with sterilized distilled water. The sterilized caryopses were cultured individually in glass culture tubes containing approximately 15 mL of 1/4 strength MS added with 0.8% agar. Cultures were maintained in a growth chamber at 25°C ± 1°C with a 16-h photoperiod under fluorescent tubes at a light intensity of 27 μmol m^–2^ s^–1^. One-week after germination, plantlets were screened for the presence of (cultivable) organism on specific media (DRBC and LB) to assess the absence of fungal or bacterial contamination.

Afterward, plants (three each jar) were placed for 14 days in a glass jar containing MS medium, spiked with AFB1. AFB1 was dissolved in an adequate amount of DMSO so that the final concentration of the solvent in culture medium did not exceed the one considered toxic (0.2%), with mycotoxin being at the final concentration of 100 μg/100 mL (absolute amount: 200 μg). Solutions were sterilized by 0.2-μm filters and dissolved in the liquid medium–containing flasks. Liquid medium without mycotoxin was used in all experiments as a control. To monitor the evolution of its absorption, AFB1 presence in liquid media was determined five times at the following intervals: *t* = 0, *t* = 1 day, *t* = 7 day, and *t* = 14 day. The experiment time was previously optimized to avoid the occurrence of visual symptoms in the control experiments (i.e., leaf senescence). After 14 days, neither leaf nor root cultures exposed to 200 μg AFB1 showed any visible degradation. All the experiments were carried out in triplicate. At the end of the experiment, above- and below-ground organs were separated, frozen in liquid nitrogen, and stored at −80°C until the analysis.

### Sample Preparation

#### Maize Sample Preparation

One gram of grounded maize sample from plants grown under field conditions was extracted by adding 4 mL of solvent mixture of acetonitrile/water/acetic acid (79:20:1, vol/vol) and stirred for 90 min at 200 strokes/min on a shaker. The extract was centrifuged for 10 min at 14,000 rpm at room temperature, and then it was passed through an immunoaffinity column (AflaStar^TM^ M1 R IAC, RomerLabs, Tunn, Austria) for clean-up. AFB1 was eluted with methanol at a rate of 1–2 drops/s and then evaporated to dryness under a stream of nitrogen. Samples were quantitatively analyzed using LC-MS.

##### Below- and above-ground organs

After 14 days of AFB1 exposure, roots and leaves of each plantlet were separately freeze dried for 12 h using a laboratory lyophilizer (LIO-5PDGT, 5Pascal s.r.l., Trezzano sul naviglio, Milano) and then milled using liquid nitrogen. Fifty milligrams of homogenized plant material was extracted by adding 1,500 μL of solvent mixture of acetonitrile/water/acetic acid (79:20:1, vol/vol) and stirred for 90 min at 200 strokes/min on a shaker. The extract was centrifuged for 10 min at 14,000 rpm at room temperature, and then 500 μL of supernatant was evaporated to dryness under nitrogen and finally resuspended in 500 μL of water/methanol (80:20, vol/vol) prior to LC-MS analysis.

##### Growing media

All medium samples were diluted with water/methanol (80:20, vol/vol) to achieve a final ratio of 1:1 (vol/vol), vortexed for 1 min, and then subjected to LC-MS analysis.

### Ultrahigh-Performance Liquid Chromatography–HRMS Screening of AFB1 Metabolites

Below- and above-ground organ extracts were subjected to HRMS in order to investigate the formation of AFB1 biotransformation products, for which analytical standards were not available.

For the chromatographic separation, a reversed-phase C18 Kinetex EVO column (Phenomenex, Torrance, CA, United States) with 2.10 × 100 mm and a particle size of 1.7 μm heated to 40°C was used. Ten microliters of sample extract was injected into the system; the flow rate was 0.3 mL/min.

Gradient elution was performed by using water (eluent A) and methanol (eluent B) both acidified with 0.5% acetic acid. Initial conditions were set at 10% B; after 3 min of isocratic step, a linear change to 90% B in 17 min; 2 min of isocratic step was followed by a reconditioning step for 5 min using initial composition of mobile phases. The total run time was 27 min.

LC-HRMS full scan spectra were recorded using Q-Exactive^TM^ high-resolution mass spectrometer (Thermo Scientific, Bremen, Germany) equipped with electrospray ionization.

The Q-Exactive mass analyzer was operated in the full MS/data-dependent MS/MS mode at the following parameters: sheath and auxiliary gas flow rates 32 and seven arbitrary units, respectively; spray voltage 3.3 kV; heater temperature 220°C; capillary temperature 250°C; and S-lens RF level 60. The following parameters were used in full MS mode: resolution 70,000 full-width half maximum (FWHM) (defined for m/z 200; 3 Hz), scan range 100–900 m/z, automatic gain control (AGC) target 3e^6^, and maximum injection time (IT) 200 ms. Parameters for dd-MS/MS mode were as follows: intensity threshold 1e4, resolution 17,500 FWHM (defined for m/z 200; 12 Hz), scan range 50—fragmented mass m/z (m/z + 25), AGC target 2e^5^, maximum IT 50 ms, and normalized collision energy (NCE) 35% with ±25% step.

Confirmation of AFM1, AFM2, AFB2, and AFL metabolites in naturally incurred maize was performed following the recommendations regarding identification using MS spectra (Guidance Document on Identification of Mycotoxins in Food and Feed, SANTE/12089/2016 implemented by 01/01/2017). When using HRMS, the precursor ion and one product ion with mass accuracy ≤5 ppm are required for the identification. In addition, the retention time of the analyte in the sample should correspond to that of the standard (tolerance ± 0.2 min).

### *In silico* Analysis

#### Model Preparation

The 3D model for human cytochrome (CYP450 1A2) was derived from the crystallographic structure recorded in the Protein Data Bank^1^ having PDB code 2HI4 (Sansen et al., 2007). The model for maize cytochrome (CYP450 81D11) was derived through homology modeling using the structure with PDB code 2HI4 as template structure, in agreement to previous studies (Dellafiora et al., 2020). The primary sequence of maize CYP was retrieved from the NCBI databank^2^. The structures were processed using the Sybyl software, version 8.1^3^, checking the consistency of atom and bond types assignment and removing the cocrystallized ligand and waters, as previously reported (Dellafiora et al., 2020).

#### Docking Simulations

Genetic Optimization for Ligand Docking (GOLD) software was chosen to perform docking studies as appropriate for computing protein–ligand interactions (Rollinger et al., 2006; Maldonado-Rojas and Olivero-Verbel, 2011). The internal GOLDScore scoring function was used as it properly assesses the contributions of protein–ligand interaction providing realistic architectures of binding (Dellafiora et al., 2020). In this respect, 25 poses for each simulation were generated, but only the best scored in each model was carried out for the analysis and considered representative of binding geometry, in agreement with previous studies (Dellafiora et al., 2020). The occupancy of the binding site was set within a sphere 10 Å around the centroid of the pocket. Proteins were set semiflexible with polar hydrogen atoms set free to rotate, whereas AFB1 was set fully flexible. The 3D structure of AFB1 was retrieved from PubChem^4^.

#### Sequence Analysis

The selection of maize cytochrome was based on the sequence identity percentage to the human CYP450 1A2. The sequence search was done through the Protein Basic Local Alignment Search (Protein BLAST) Tool of the NCBI data bank^5^ using the FASTA sequence of the PDB structure 2HI4 as input sequence and limiting the search to *Z. mays* (taxid:4577) sequences.

## Results

### Evidence of the Occurrence of Modified Aflatoxins in Field-Grown Maize

Our primary goal was to evaluate whether the main hydroxylated aflatoxin metabolites could occur in maize naturally infected by *Aspergillus* spp. fungi. At this purpose, we selected from our in-house sample collection several maize samples already known from previous studies to be contaminated by AFB1 under field conditions (Righetti et al., 2020, personal communication). These samples were analyzed by LC-HRMS for the possible co-occurrence of AFB1, AFB2, AFG1, AFG2, and the main hydroxylated forms AFM1, AFM2, and AFL. Sample extracts were run by dilute-and-shoot. Data are reported in Table 1.

**TABLE 1:** Occurrence of aflatoxins in selected maize samples.

Sample		Mycotoxins (μ g kg ^–1^)						
		
		AFB1	AFB2	AFG1	AFG2	AFM1	AFM2	AFL
ZM1	Mean	128	4	<LOD	<LOD	9	2	<LOQ
	SD	2	1.1	—	—	1.7	0.8	—
ZM2	Mean	139	10	<LOD	<LOD	10	8	<LOD
	SD	5.4	0.4	—	—	0.2	0.4	—
ZM3	Mean	63	7	<LOD	<LOD	6	<LOD	<LOQ
	SD	1.3	0.5	—	—	0.8	—	—
ZM4	Mean	47	8	22	<LOD	2	<LOD	<LOD
	SD	2.1	0.1	0.1	—	0.1	—	—
ZM5	Mean	28	1	<LOD	<LOD	2	<LOD	<LOD
	SD	2.5	0.2	—	—	0.3	—	—
ZM6	Mean	28	2	<LOD	<LOD	2	<LOD	<LOD
	SD	1.1	0.1	—	—	0.1	—	—
ZM7	Mean	24	5	<LOD	<LOD	3	<LOD	<LOD
	SD	0.4	0	—	—	0.2	—	—
ZM8	Mean	23	5	12	<LOD	2	<LOD	<LOD
	SD	2.3	0.8	0	—	0.3	—	—
ZM9	Mean	14	<LOQ	<LOD	<LOD	1	<LOD	<LOD
	SD	1.8	—	—	—	0.4	—	—
ZM10	Mean	14	<LOQ	<LOD	<LOD	1	<LOD	<LOD
	SD	3.7	—	—	—	0.3	—	—
ZM11	Mean	17	<LOQ	<LOD	<LOD	1	<LOD	<LOD
	SD	1.9	—	—	—	0.2	—	—
ZM12	Mean	11	<LOQ	<LOD	<LOD	0.5	<LOD	<LOD
	SD	1.8	—	—	—	0.2	—	—
ZM13	Mean	<LOD	<LOD	<LOD	<LOD	<LOD	<LOD	<LOD
	SD	—	—	—	—	—	—	—
ZM14	Mean	<LOD	<LOD	<LOD	<LOD	<LOD	<LOD	<LOD
	SD	—	—	—	—	—	—	—
ZM15	Mean	<LOD	<LOD	<LOD	<LOD	<LOD	<LOD	<LOD
	SD	—	—	—	—	—	—	—

*LOD (limit of detection): AFB1 0.025 μg kg^–1^, AFB2 0.025 μg kg^–1^, AFG1 0.25 μg kg^–1^, AFG2 0.25 μg kg^–1^ and AFM1 0.05 μg kg^–1^. LOQ (limit of quantification): AFB2 0.25 μg kg^–1^. Data are reported as mean ± standard deviation of three replicates. Samples are listed according to an internal coding system.*

Surprisingly, AFM1 occurred in 12 of 15 samples, and AFM2 was found in two samples, both at μg/kg levels. On the contrary, AFL was clearly detected but at non-quantifiable levels in two samples. Compared to AFB1 concentration, the AFM1 percentage was in the range 3.6–13.1%, with a mean value of 7.6%. In contrast, AFM2 concentration was comparable to AFB2 and ranging between 1.7 and 5.8% compared to AFB1.

**FIGURE 1:**
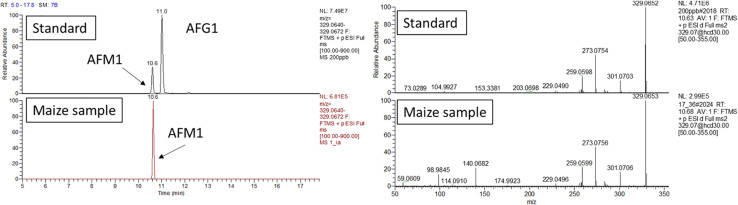
AFM1 confirmation analysis. Retention time and HRMS/MS comparison between calibration standard and maize extract analyzed in the same sequence. The maize extract was passed through an immunoaffinity column for selective clean-up. UHPLC-Q-Exactive full scan (on the left) extracted ion chromatogram (resolving power 70,000 FWHM, extraction window 5 ppm) and (right side) fragmentation pattern obtained with a collision energy of 30 eV.

All the annotated peak compounds passed all the requirements described in the “Materials and Methods section,” and therefore, they were univocally identified as AFM1, AFM2, and AFL (see Figure 1 for AFM1 and Supplementary Material for other metabolites).

As additional confirmation, maize extract underwent immunoaffinity clean up. This step allowed for an high specificity toward AFM1, which was concentrated and further confirmed.

### Evidence of AFB1 Uptake and Biotransformation in *Z. mays* L. Plants *in vitro*

To gain more insights into the ability of maize to biotransform AFB1 into its Phase I metabolites, the model assay already in use in our laboratory and based on *in vitro*–grown plantlets was adopted (Righetti et al., 2017, 2020). AFB1-treated and control plantlets were grown for 14 days.

The administered mycotoxin was not detected in the blank medium or in roots and leaves blanks. In addition, no degradation of AFB1 occurred because of chemical and physical agents (medium constituents and pH, temperature, and light) during the whole experiment. No visual symptoms of phytotoxicity were observed in roots and leaves over the treatment observation time.

AFB1 uptake from the medium was monitored over time, as well as the extraction of modified forms. As for the kinetic reported in Table 2, the plant absorption was almost complete after 14 days, with only 2% of residual AFB1 in the medium.

**TABLE 2:** Absorption of AFB1 from the growing medium (initial amount: 200 μg).

Time point	AFB1 mean ± SD [%]
t0	1000
t24h	742.3
t7d	110.1
t14d	20.1

*Data are given in terms of residual AFB1% in the medium (*n* = 3) (time points: 0, 24 h, 7 days, and 14 days).*

After 14 days of incubation, above- and below-ground organs were separately analyzed by LC-HRMS for aflatoxin B1 and its modified forms, with the aim of investigating not only the possible root absorption, but also the potential translocation and biotransformation of aflatoxin B1 in different plant organs. Metabolites have been annotated based on the MS/MS fragmentation and univocally identified by authentic reference compound injection, as reported in Table 3.

**TABLE 3:** Phase I metabolites of AFB1 annotated from roots and leaves analysis and their qualitative abundance.

Metabolite	logP	RT (min)	Formula	Adduct	Theoretical *m/z*	Mass error (ppm)	Leaves/roots
AFM2*	0.2	9.7	C17H14O7	[M + H]^+^	331.0812	–0.8	0.09
AFQ1	0.5	9.8	C17H12O7	[M + H]^+^	329.0656	–1.3	0.55
AFM1*	0.5	10.3	C17H12O7	[M + H]^+^	329.0656	0.6	2.23
AFP1	1.3	10.3	C16H10O6	[M + H]^+^	299.0550	–1.6	0.01
AFB2*	1.3	10.9	C17H14O6	[M + H]^+^	315.0863	–0.8	0.06
AFB1*	1.6	11.4	C17H12O6	[M + H]^+^	313.0707	–0.8	0.08
AFL*	1.2	12.4	C17H14O6	[M-H_2_O + H]^+^	297.0757	–1.5	0.09

*Confirmation with standard by comparison of accurate mass, HRMS/MS, and RT. All the metabolites were detected in both organs. The abundance, expressed as the peak chromatographic area, was always higher in roots than in leaves for all the annotated metabolites with the only exception of AFM1.

Besides the administered parent compound, AFB2, AFL, aflatoxin M1, and aflatoxin M2 were found in both roots and leaves, clearly suggesting an extensive Phase I metabolism (see Supplementary Material). The same metabolites were found in the medium, actually indicating a possible release by roots due to the increased polarity. Moreover, AFP1 and AFQ1 have been detected at trace levels in roots and leaves.

The initial annotation was performed based on accurate MS and HRMS/MS spectra for all the considered compounds. As already done for the analysis carried out on naturally incurred maize, reference standards were used for the univocal identification when available.

### *In silico* Evaluation of the Interaction Between Aflatoxin B1 and *Z. mays* L. Cytochrome

**FIGURE 2:**
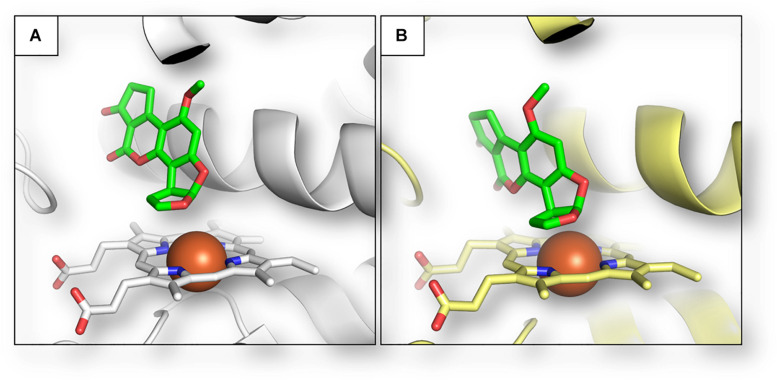
Calculated binding architecture of AFB1 within human CYP450 1A2 **(A)** and maize CYP450 81D11 **(B)**. Proteins are shown in cartoon, while AFB1 and heme group are represented in sticks. Red spheres indicate the iron ions.

To confirm the role of plant metabolism in AFB1 biotransformation, we considered whether *Z. mays* cytochromes could be able to metabolize such mycotoxin into its already known hydroxylated forms. To do so, the putative interaction between AFB1 and plant cytochrome was compared to that of human CYP450 1A2, which is one of the isoforms predominantly converting AFB1 to AFM1 (Bonomo et al., 2017). As shown in Figure 2, the calculated pose of AFB1 within the human CYP450 1A2 provided a consistent binding architecture, with the difuran moiety well oriented toward the heme group to receive oxidation and to form hydroxylated metabolites such as AFM1 (Figure 2A). Although there are no available crystallographic structures of AFB1 bound to this cytochrome to geometrically validate the model, this finding supported the reliability of the approach used to provide an accurate binding pose of AFB1 considering the plausible binding pose obtained. The interaction between AFB1 and the maize cytochrome CYP450 81D11 was then calculated. This specific isoform was selected to exploratory assess the capability of plant cytochromes to produce AFB1 metabolites (e.g., AFM1) likewise human cytochromes. Specifically, CYP450 81D11 was chosen as it showed the highest sequence identity (30.65%) among the other maize cytochromes identified so far compared to the human CYP450 1A2 (last database access October 19, 2019). This feature could reasonably determine a degree of functional conservation to produce an array of metabolites similar to the human CYP 1A2. As shown in Figure 2B, the interaction of AFB1 within the plant cytochrome model mirrored the interaction observed within the human CYP450 1A2, pointing to the possible functional conservation in terms of metabolite production of the two orthologs.

## Discussion

To cope with exogenous and potentially deleterious compounds of different origin, every living organism has evolved specific defensive strategies. One of the most effective relies on enzymatic pools as cytochrome P450 monooxygenases or glutathione *S*-transferases, whose presence and detoxifying activity are widespread in any phyla and kingdom (Gonzalez and Nebert, 1990). While such evidence is firmly rooted among biologists and ecologists, it seems to be somewhat neglected when it comes to risk assessment of mycotoxins in feed and food. The fact that previous detection of AFM1 in plants was explained by food toxicologists with weak hypothesis involving fungal, bacterial, or insect metabolism, ignoring an active role of vegetables, seems to confirm that. Our data confirm instead that plants have an active role in the production of hydroxylated aflatoxins and suggest that plant cytochromes may be involved in the biotransformation of AFB1 into AFM1, AFM2, and AFL. Results were obtained by multiple and convergent experiments on field-grown plants, *in vitro* grown plantlets, and by *in silico* evaluations, each one offering interesting insights.

First, data presented herein clearly demonstrate that AFM1 and other Phase I hydroxylated metabolites of AFs may occur in naturally incurred maize samples; AFM1, AFM2, and AFL have been identified and quantified at a percentage up to 13% compared to AFB1. These results suggest that in highly contaminated samples the presence of AFM1 should not be overlooked. At the same time, such evidence must be placed in the proper context. It is in fact relevant to underline that the naturally incurred samples considered in this study were selected with the purpose to maximize the possible occurrence of modified forms (AFB1: 10–140 μg/kg). Therefore, these samples were already not compliant with the current EU regulation for human consumption and dairy feed (EU Maximum Residue Levels (MRL) for AFB1 in maize: 5 μg/kg), even before including the contribution of its hydroxylated forms.

Since maize samples considered within this study have been grown and harvested under open field conditions, an AFB1 biotransformation related to microbial activity of exogenous or of endophytic origin and occurring preharvest or postharvest cannot be ruled out. Nevertheless, the hypothesis of a Phase I metabolism exerted by plants on AFs is worth of investigation, being such modifications already known for other mycotoxins with different structures (Meng-Reiterer et al., 2015; Nathanail et al., 2015; Righetti et al., 2017, 2019).

As data from naturally incurred samples do not provide an unequivocal confirmation of an active role of plants in aflatoxin biotransformation, we performed further experiments on *in vitro*, axenic cultures of maize plantlets. While results may differ from those observed in open field conditions and with fully developed plants, our model offers major advantages in terms of standardization and control of variables involved.

Our *in vitro* experiment clearly demonstrates the ability of maize plantlets to uptake and convert AFB1 into AFB2, together with a range of hydroxylated forms, even when a pathogenic attack is not actually occurring. As a confirmation of the involvement of maize Phase I metabolism, AFM2 and AFL were found to be the most abundant metabolites in both roots and leaves, suggesting a major role played by Phase I reduction compared to Phase I hydroxylation in maize. The occurrence of AFM1 together with the isomeric AFP1 and AFQ1 was also observed at a lower extent. The relative abundance of AFB1 and its modified forms was, as expected, higher in roots than in leaves, confirming that the biotransformation immediately follows the uptake from the growth medium. This is in agreement with previous studies reporting on the high metabolic activity and the relevant enzymatic potential observed in maize roots (Dixon et al., 1997). However, AFB1 was found in leaves too, thus demonstrating its translocation to above-ground organs. Interestingly, all the modified forms were more abundant in roots than in leaves, with the only exception of AFM1. Its comparable abundance in the two organs may suggest a preferred organ-specific metabolism or a higher translocation rate of the already formed AFM1. When suggesting an unspecific translocation mechanism, it should be noticed that AFM1 and other isomeric hydroxylated forms share a comparable solubility, as attested by comparable logP values. Therefore, a differential translocation from roots is unlikely in favor of a more efficient biotransformation of AFB1 already translocated in leaves. It should be made clear, however, that the findings presented here were obtained in a simplified system. For instance, it remains to be examined if and how such transformation and the described translocation take place in full-grown plants, under field conditions and in particular when the exposure to AFB1 occurs *via Aspergillus* infection in ears rather than from radical uptake of aflatoxins.

**FIGURE 3:**
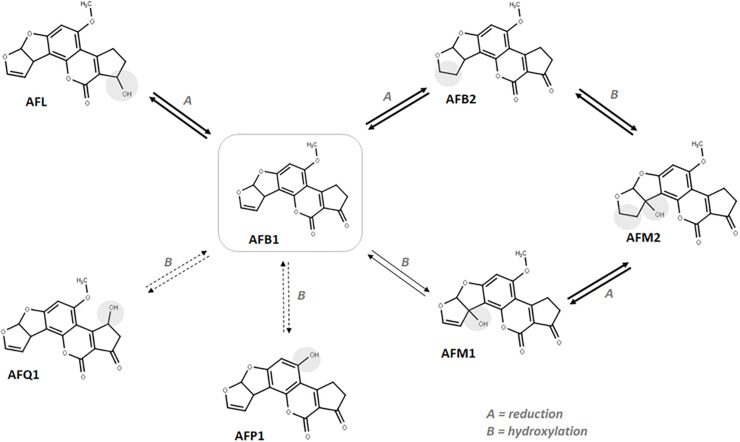
Suggested *in vitro* biotransformation occurring to AFB1 in maize. Darker arrows indicate the most abundant metabolites in roots. Dotted arrows indicate the less accumulated metabolites in roots.

These results confirm the capability of maize plantlets to autonomously produce hydroxylated AFs by simple chemical exposure to AFB1 and strengthen the hypothesis that plant-detoxifying enzymes may be responsible for such biotransformation. In consideration of the evidence provided, a tentative scheme of the possible occurring biotransformations is presented in Figure 3.

The higher accumulation of AFM2 compared to other metabolites suggests that reduction may occur quicker than CYP-mediated hydroxylation, thus favoring the AFB1-AFB2-AFM2 biotransformation route. Similarly, once formed, AFM1 could be quickly reduced to the more polar AFM2. However, observing the leaf-to-root abundance ratio reported in Table 3, the inversion reported for AFM1 compared to other metabolites suggests an organ-specific metabolism, likely due to a lower reduction rate that leads to an accumulation of AFM1 by decreasing the AFB1 to AFB2 as well as AFM1 to AFM2 conversion efficacy. According to the data collected, the ratio between aflatoxin B1 and its modified forms is rather low (<8% in average). Therefore, as explained for naturally incurred samples, the possible co-occurrence of hydroxylated metabolites is unlikely to pose any significant health concern also considering an eventual radical uptake from soil by healthy plants.

Ideally, a xenobiotic compound is metabolized to its more hydrophilic form through Phase I and Phase II metabolism and finally sequestered in vacuoles or cell walls. Phase I reactions are initiated by cytochrome P450 complex leading to oxidation, reduction, or hydroxylation of xenobiotics. Therefore, to suggest a potential path for further investigations, we have finally performed an *in silico* evaluation of *Z. mays* L. cytochromes to exert Phase I metabolism on aflatoxin B1. To note, CYP450 1A2 was described as one of the isoforms most involved in AFM1 production in humans. Therefore, from a toxicodynamic perspective, it could be inferred that plants may produce hydroxylated metabolites, including AFM1, similarly to human orthologs. Nonetheless, kinetics aspects need to be further investigated as differences in kinetics of transformation may be crucial to differentiate the yield of formation of hydroxylated metabolites in living organisms, with important consequence on both toxicity and risk assessment of AFB1. In this respect, a large-scale modeling of toxicodynamic and toxicokinetic parameters of plant-metabolizing enzymes may support a systematic analysis to support the identification of those most likely involved in AFB1 transformation. This approach could provide a sound line of evidence either to improve the current risk assessment or to better understand the mechanistic aspect of the strategies used by plant to tackle mycotoxins accumulation.

As far as the relevance of our findings for risk assessment, it has to be noted that the amount of AFM1 found in maize is significant, compared to the current legislation, only when AFB1 content is far above the MRLs. Therefore, it can be assumed that the metabolic conversion in plants likely leads to negligible amounts of AFM1 in compliant samples. However, as aflatoxins are well-known carcinogenic compounds, the ALARA (as low as reasonably achievable) principle should be applied. Collecting knowledge about any possible form responsible for adverse effects *in vivo* is therefore relevant for food toxicologists.

Overall, these results may suggest more than a simple experimental confirmation or a new evidence. In an applied science as food toxicology, complexity must be always embraced: multiple transdisciplinary approaches must be always welcomed and enforced, providing new hypothesis and challenging previous paradigms. As Brady et al. (2017) suggested, in toxicology, comprehensive investigations involving multiple disciplines generate basic understanding that informs both screening efforts, risk assessment, and regulatory decision-making. However, some points of view are somewhat neglected, whereas other establish themselves more firmly.

For instance, within the field of food mycotoxins, we have focused in recent years on the understanding of the defensive pathways activated by the plant toward mycotoxins, mostly describing the latter as virulence factors or phytotoxic compounds. Plants, on the other hand, seem to handle any mycotoxin as a common xenobiotic, reacting to any exposure with the activation of the same non-specific biochemical machinery operational and already described for many manmade chemicals, i.e., drugs and pesticides. While providing enormous levels of information and knowledge, an exclusive recourse to such interpretation may limit the evaluation and interpretation of food toxicologists.

As for any other phenomena regarding living organisms, a finalistic purpose has to be excluded. Plants seem, in fact, to undergo the biotransformation route for any xenobiotic compound that may be modified by their enzymatic pools, whether the “original role” of these compounds is to cause harm or not. Furthermore, as with any biological system, not a single solution is followed but rather a combination of several solutions joined to form a strategy, in order to maximize the resilience of the whole system.

It must be also considered on this regard that a possible role of AF in fungal virulence has yet to be confirmed or ruled out, and these substances could be involved in the toxic interplay between plants and fungi in ways that we do not know at present.

The scientific gap in respect to the possible formation of masked aflatoxins is partly due, in our opinion, to the great emphasis placed on the analytical performance rather than on the real understanding of the biochemical phenomena occurring at a molecular level and on the involvement of plants biochemical machinery. As a matter of fact, it has led to a sort of analytical blindness that can be distilled in a simple assumption: if the modified aflatoxins are never sought in the plant, they will surely not be found. Plant Phase I metabolites of AFB1 were always present in maize and were spotted seldom in the past in other plants, but no one looked for them or explained their presence because they were firmly related to animal metabolism.

This work, therefore, not only provides the first evidence about the ability of maize to effectively biotransform AFB1 into its Phase I modified forms, but also—above all—emphasizes the need for a paradigm shift in mycotoxin research, to ensure a comprehensive risk assessment under the guidance suggested by Brady et al., 2017 in 2017: “Toward a unified understanding of life’s response to toxic chemicals.”

## Data Availability Statement

The datasets presented in this study can be found in online repositories. The names of the repository/repositories and accession number(s) can be found below: Mendeley Repository, DOI: 10.17632/mx3j8ncb5y.2 https://data.mendeley.com/datasets/mx3j8ncb5y.2.

## Author Contributions

LR, RB, and CD’A conceived the study. ER planned and carried out the experiments on plant materials. LD planned and carried out the *in silico* modeling. LR carried out the HR-MS analysis and the data elaboration. MS and GG contributed to the interpretation of the analytical results. LR and RB equally contributed to the writing process. GG, MS, and CD’A critically revised the final manuscript. CD’A supervised the project. All authors provided critical feedback and helped shape the research.

## Conflict of Interest

MS is employed by the company Barilla G.R. F.lli SpA, Italy. The remaining authors declare that the research was conducted in the absence of any commercial or financial relationships that could be construed as a potential conflict of interest. The reviewer MR declared a past collaboration with one of the authors LD to the handling editor.

## Footnotes

1 https://www.rcsb.org

2 https://www.ncbi.nlm.nih.gov; accession code XP_008661335.

3 www.certara.com

4 https://pubchem.ncbi.nlm.nih.gov; Compound CID: 186907.

5 https://blast.ncbi.nlm.nih.gov/Blast.cgi
