# Supplementary material for: Thinking Out of the Box: On the Ability of Zea mays L. to Biotrasform Aflatoxin B1 Into Its Modified Forms
Source: Front Plant Sci. 2021 Jan 12;11:599158. doi: 10.3389/fpls.2020.599158 (PMC7835335; doi:10.3389/fpls.2020.599158)
Supplement: Supplementary file 1 [file Table_1.DOCX]

Supplementary Material

Thinking out of the box: on the ability of *Zea Mays* L. to biotrasform aflatoxin B1 into its modified forms.

Laura Righetti^1*^, Enrico Rolli^2^, Luca Dellafiora^1^, Gianni Galaverna^1^, Michele Suman^3^, Renato Bruni^1^, Chiara Dall’Asta^1*^

^1^Department of Food and Drug, University of Parma, Viale delle Scienze 17/A, I-43124 Parma, Italy

^2^Deparment of Chemistry, Life Sciences and Environmental Sustainability, University of Parma, Via G.P. Usberti 11/a, Parma, Italy

^3^Barilla G.R. F.lli SpA, Advanced Laboratory Research, via Mantova 166, Parma, Italy

*** Correspondence:**Laura Righetti: [laura.righetti@unipr.it](mailto:laura.righetti@unipr.it)

Chiara Dall’Asta: [chiara.dallasta@unipr.it](mailto:chiara.dallasta@unipr.it)


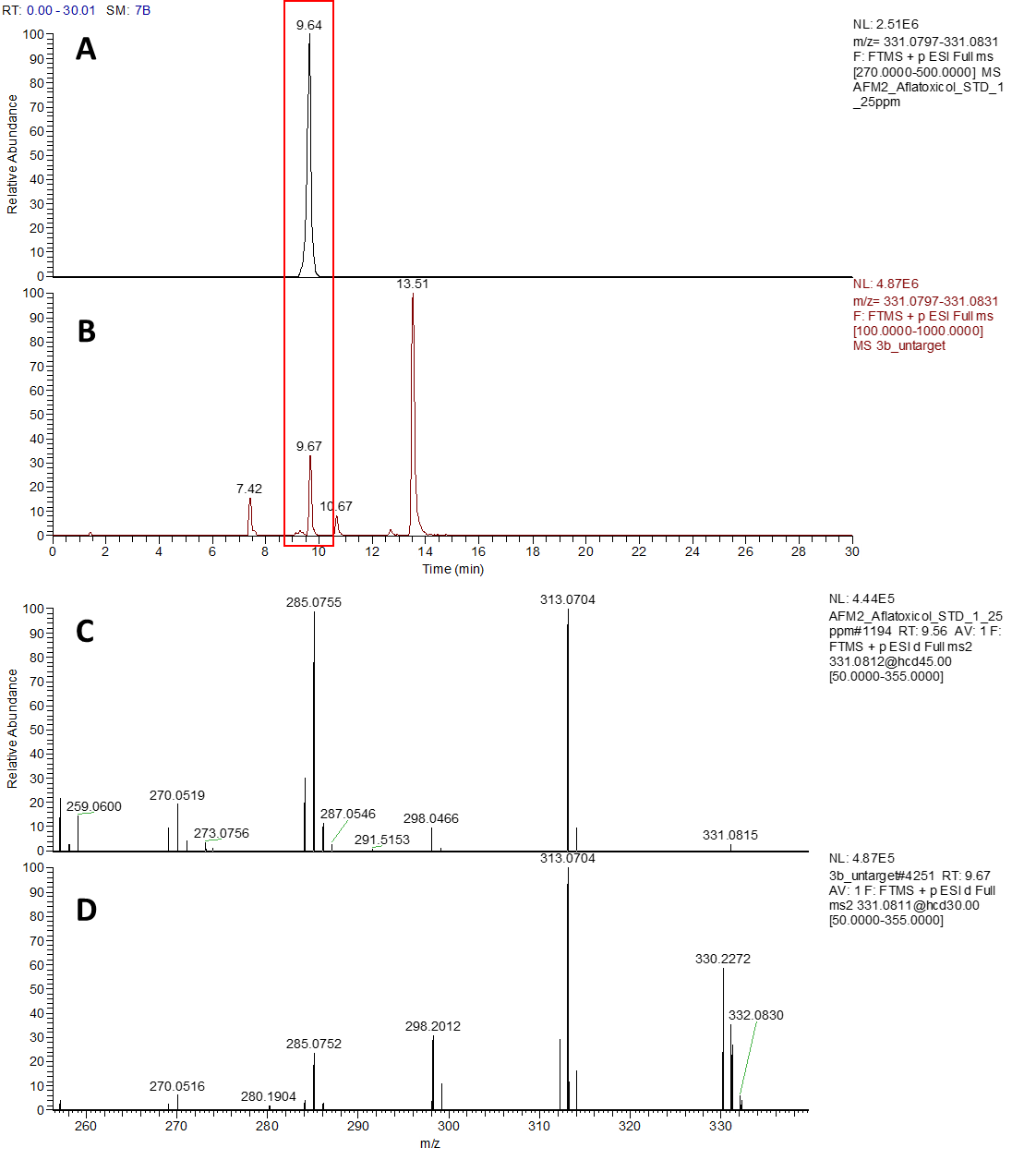


**Supplementary Figure 1.** UHPLC-HRMS/MS spectrum of aflatoxin M2.

UHPLC – Q Exactive full scan extracted ion chromatogram (resolving power 70,000 FWHM, extraction window 5 ppm) of AFM2 [M+H]^+^ m/z 331.0814 in AFM2 standard (A) and AFB1-treated maize sample (B). High-resolution fragmentation spectrum, obtained by using DDA acquisition, of AFM2 [M+H]^+^ m/z 331.0814 in AFM2 standard (C) and AFB1-treated maize root (D).

**
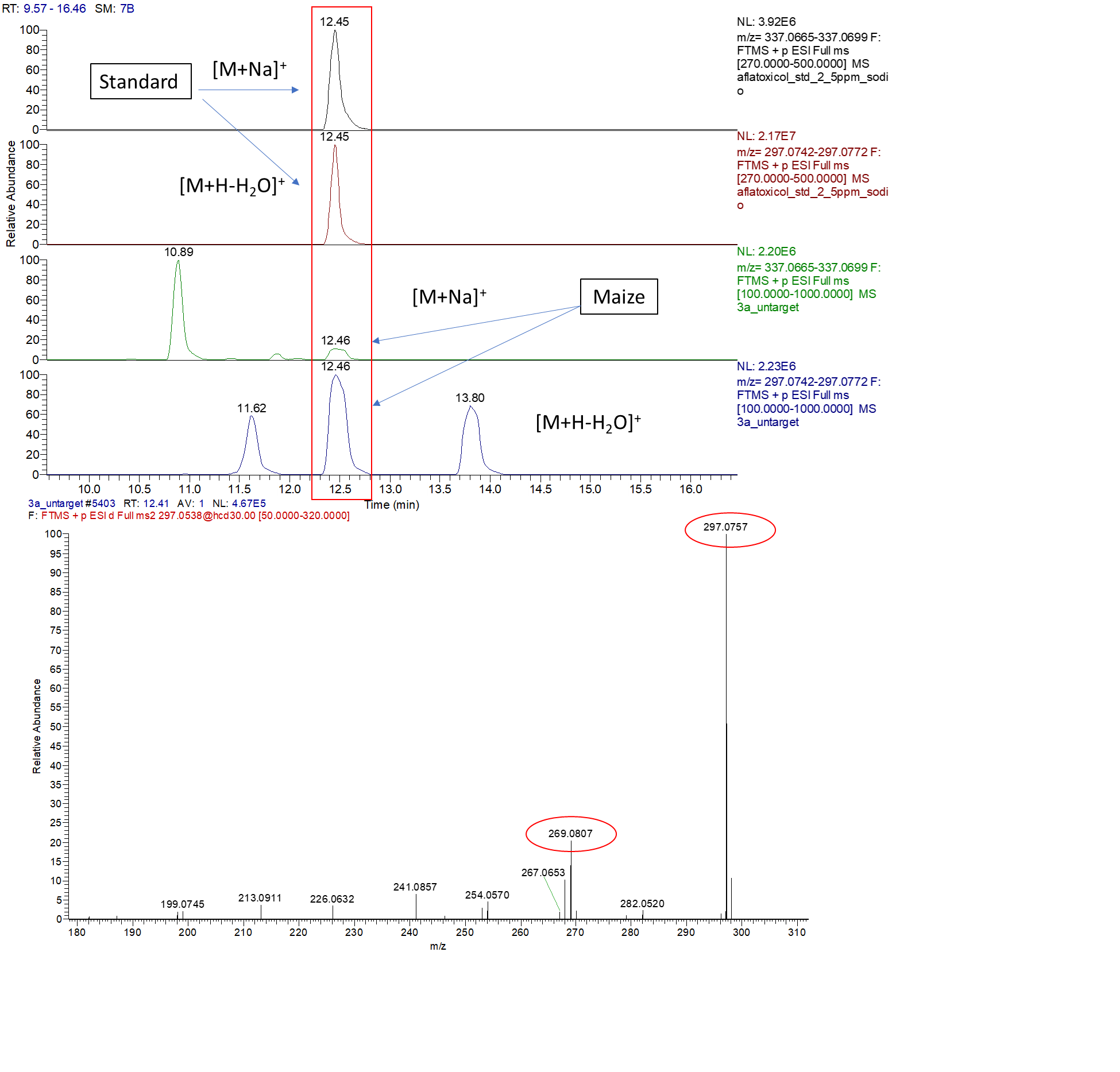
**

**B**

**A**

**Supplementary Figure 2.** UHPLC-HRMS/MS spectrum of aflatoxicol.

Retention time and HRMS/MS comparison between calibration standard and maize extract analyzed in the same sequence. UHPLC-Q-Exactive full scan (A) extracted ion chromatogram (resolving power 70,000 FWHM, extraction window 5 ppm) and B) fragmentation pattern obtained with a collision energy of 30 eV. Aflatoxicol was detected both as sodium adduct ([M+Na]^+^ *m/z* 337.0682) and as water ion ([M+H-H_2_O]^+^ *m/z* 297.0757), with the latter giving the highest intensity.


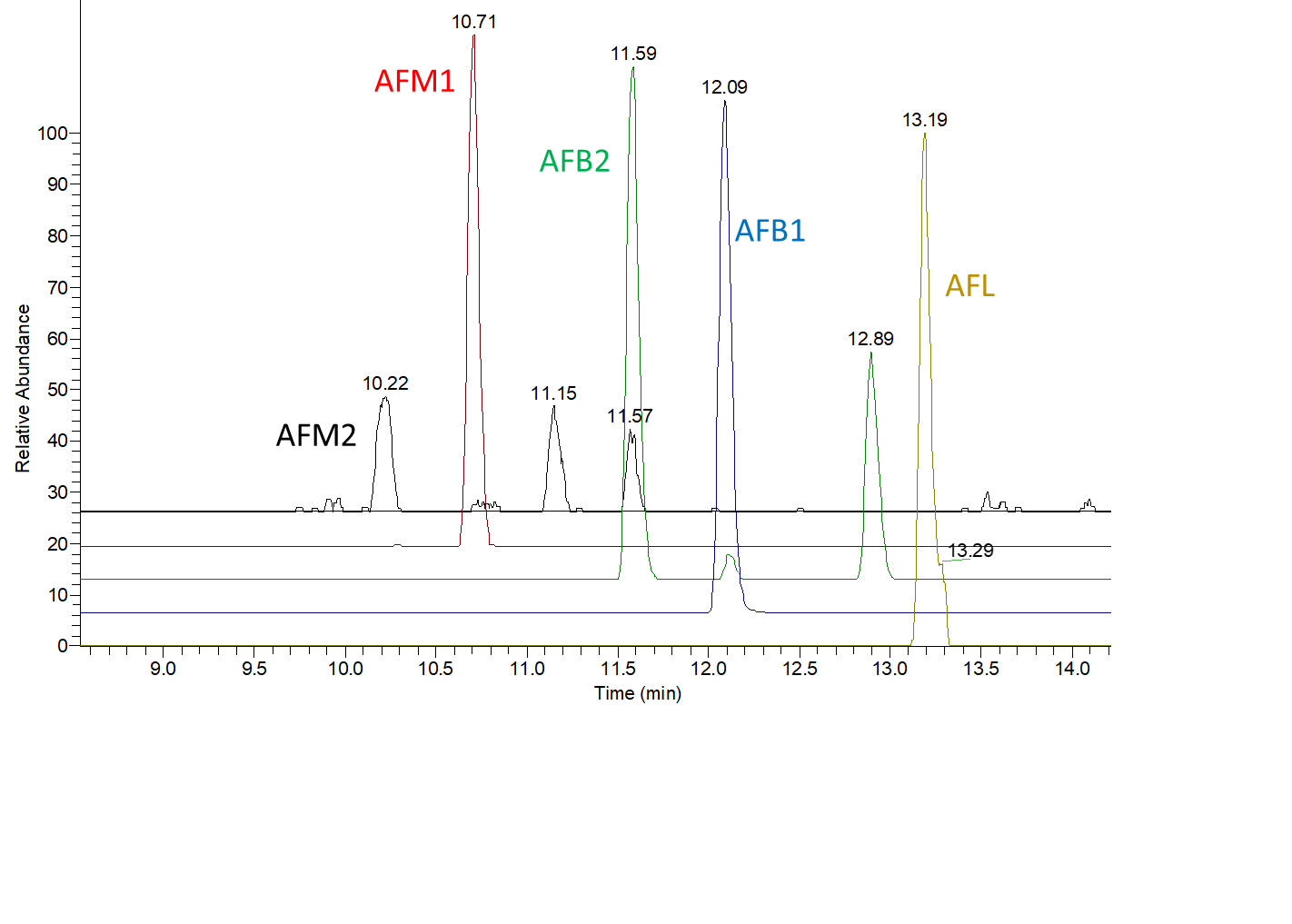


**Supplementary Figure 3.** UHPLC-Q-Exactive full scan extracted ion chromatogram (resolving power 70,000 FWHM, extraction window 5 ppm) of maize sample (#ZM1) naturally contaminated by AFB1, AFB2, AFM1, AFM2 and AFL.
